# Metabolomic biomarkers in vitreous humor: unveiling the molecular landscape of diabetic retinopathy progression

**DOI:** 10.1186/s40942-025-00682-5

**Published:** 2025-05-22

**Authors:** John Kim Hiller, Elise Mørk Sandås, Helge Rootwelt, Anja Østeby Vassli, Xhevat Lumi, Morten Carstens Moe, Tor Paaske Utheim, Katja Benedikte Prestø Elgstøen, Goran Petrovski

**Affiliations:** 1 Center for Eye Research and Innovative Diagnostics, Department of Ophthalmology, Institute of Clinical Medicine, Faculty of Medicine, Oslo University Hospital, University of Oslo, Kirkeveien 166, Oslo, 0450 Norway; 2 Department of Medical Biochemistry, Oslo University Hospital, Oslo, Norway; 3 Department of Ophthalmology, Justus-Liebig-University Giessen, University Hospital Giessen and Marburg GmbH, Giessen, Germany; 4 Department of Ophthalmology, Sørlandet Hospital, Arendal, Norway; 5 Department of Ophthalmology, University Hospital Centre, University of Split School of Medicine, Split, Croatia; 6 UKLO Network, University St. Kliment Ohridski, Bitola, North Macedonia

**Keywords:** Diabetic retinopathy, Metabolomics, Vitreous humor, Biomarkers, HRMS, HPLC

## Abstract

**Background:**

Diabetic retinopathy (DR) is a progressive retinal disease that leads to vision loss if not detected early. Metabolomic analysis of vitreous humor offers a promising approach to identifying biomarkers associated with disease onset and progression. This pilot study investigates the metabolomic profiles of vitreous humor from patients at different stages of DR, aiming to uncover potential biomarkers for early detection and monitoring of disease progression.

**Methods:**

Vitreous samples were collected during therapeutic pars plana vitrectomy of 23 patients without diabetes (CTRL), with diabetes and without retinopathy (DIA), non-proliferative DR (NPDR) and proliferative DR (PDR). Metabolomics was performed using high-performance liquid chromatography coupled with high-resolution mass spectrometry.

**Results:**

Principal component analysis revealed distinct metabolic signatures differentiating the patient groups. Lysine, proline, and arginine levels progressively increased from DIA to NPDR and PDR stages, highlighting their association with disease progression. Methionine and threonine showed notable increases in PDR compared to all other groups, while carnitine, a key metabolite in lipid metabolism, exhibited stage-specific increases, peaking in PDR. The detection of systemic and topical drugs, including metformin and tropicamide, in the vitreous further emphasizes altered ocular permeability in DR.

**Conclusion:**

Our findings suggest that metabolomic profiling could provide valuable insights into the underlying pathogenesis of DR and serve as a foundation for personalized therapeutic strategies.

**Supplementary Information:**

The online version contains supplementary material available at 10.1186/s40942-025-00682-5.

## Introduction

Diabetic retinopathy (DR), a microvascular complication that arises from prolonged diabetes mellitus, remains a pressing global health challenge, exacting a substantial toll on visual acuity and quality of life [1–5]. As researchers strive to untangle the molecular underpinnings of DR progression, the identification of robust biomarkers capable of distinguishing distinct stages of the disease has emerged as a pivotal pursuit [6, 7]. In this context, the growing field of metabolomics offers a promising avenue, leveraging the dynamic interplay and effects of small molecules to illuminate the metabolic signatures associated with varying stages of DR [8].

The vitreous humor, a gel-like substance occupying the posterior segment of the eye, encapsulates metabolites sourced from both local ocular tissues and systemic circulation [9]. The metabolome, a dynamic reflection of cellular processes, bears the potential to unveil the biochemical nuances characterizing different stages of DR [10]. We hypothesize that the metabolomic fingerprint present within the vitreous environment possibly holds the key to unlocking molecular insights crucial for accurate disease stratification, prognosis, and the tailoring of personalized therapeutic interventions [8, 10].

Our study sought to explore the metabolome of the vitreous, with the aim of unveiling distinctive metabolic biomarkers that demarcate patients without diabetes (CTRL), diabetes without retinopathy (DIA), non-proliferative DR (NPDR) and proliferative DR (PDR). Employing cutting-edge techniques such as high-performance liquid chromatography (HPLC) coupled with high-resolution mass spectrometry (HRMS), we analyzed the vitreous samples of patients at varying disease stages [11]. The discernment of unique metabolic fingerprints not only augments our comprehension of the underlying pathogenic mechanisms, but also paves the way for the identification of potential biomarkers that could optimize clinical decision-making and therapeutic strategies [8, 11]. With a focus on the metabolite markers pinpointed by our analysis, we examine the clinical potential of these molecular signposts in facilitating accurate stage differentiation. By bridging the gap between laboratory research and clinical application, the exploration of metabolomic biomarkers may hold the promise to contribute to the role of precision medicine in ocular health.

Previous publications discuss the comparison between the vitreous metabolomes of patients without diabetes and patients with PDR [12–17]. To our knowledge, no study comparing the vitreous metabolomes of patients without diabetes, patients with diabetes and without retinopathy, patients with NPDR and patients with PDR has been conducted. In the present paper, we present the design, methodology, and results of our comparative analysis of vitreous metabolomics in controls and patients with different stages of diabetic retinopathy. We discuss the implications of our findings in the context of existing knowledge and explore potential avenues for future research and clinical translation. The insights gained from this study will contribute to increased understanding of the pathophysiological mechanisms underlying the progression from no DR, via NPDR to full-blown PDR. Moreover, these discoveries could potentially pave the way for the development of innovative diagnostic tools and therapeutic targets, thereby contributing to the enhanced prevention and management of diabetic retinopathy and its associated complications.

## Patients and methods

### Patients

Patients undergoing therapeutic pars plana vitrectomy (PPV) at the Department of Ophthalmology, Oslo University Hospital (OUH), Oslo, Norway and the Eye Hospital, University Medical Centre, Ljubljana, Slovenia, were prospectively recruited to this pilot study. All patients underwent a clinical review with collection of medication lists at the time of inclusion, and samples were collected between February and August, 2021. Inclusion criteria: patients undergoing therapeutic PPV aged ≥ 50 years. Exclusion criteria: history of uveitis, retinal vascular occlusion, advanced glaucoma, intraocular surgery within three months preoperatively (except cataract), ongoing systemic infections, active systemic inflammatory conditions and active vitreous hemorrhage.

The samples were obtained from the following patient groups: control/non-diabetic (CTRL) (*n* = 6), diabetic without DR (DIA) (*n* = 5), NPDR (*n* = 5), and PDR (*n* = 7). The indications for vitrectomy in all patients were macular hole, epiretinal membrane, symptomatic vitreomacular traction or non-clearing vitreous opacities (Table S1). Most diabetic patients had well-regulated hypertension and dyslipidemia. One patient in the CTRL group and one patient in the DIA group had controlled open-angle glaucoma. The three patients undergoing anti-VEGF treatment received injections 6–14 days before surgery. Consultant ophthalmologists (G.P., X.L.) determined indications for vitrectomy and the level of DR. (NPDR: from presence of microaneurysms, dot- or blot- like hemorrhages, or exudates; PDR: presence of neovascularization of the optic disc or elsewhere or preretinal bleeding(s), vitreous bleeding, preretinal fibrosis, and tractional retinal detachment). The clinical characteristics of the included patients are summarized in Table 1.

**Table 1:** Clinical characteristics of the study population

Characteristic	CTRL	DIA	NPDR	PDR
Number	6	5	5	7
Age (years; mean ± SD)	77.8 ± 12.9	71.4 ± 5.1	73.6 ± 5.7	62.7 ± 3.6
Gender (male/female)	5/1	3/2	4/1	2/5
DM type (type 1/type 2)	0/0	0/5	1/4	1/6
DM duration (years; mean ± SD)	-	9.5 ± 0.7	17.6 ± 9.9	27 ± 9.5
Photocoagulation (n)	-	-	0	5
Anti-VEGF injection (n)	-	-	-	3
Pseudophakic	5	4	5	6

*CTRL = controls without diabetes or retinopathy; dia = patients with diabetes and no retinopathy; npdr = patients with non-proliferative diabetic retinopathy; pdr = patients with proliferative diabetic retinopathy; dm = diabetes mellitus; vegf = vascular endothelial growth factor*

### Ethics

All patients provided written informed consent for the collection of vitreous samples and clinical data prior to surgery. Patient samples and clinical patient data were handled in accordance with the tenets of the Declaration of Helsinki. The research protocol was approved by the Regional Committees for Medical and Health Research Ethics in Norway (REK no. 8050).

### Sample collection

Undiluted vitreous samples were collected during standard 25G PPV, before infusion was initiated. The samples were taken with the vitrector from the mid-vitreous approximately 10 mm from the retina. Depending on the ocular conditions, between 0.2- and 1.2-mL undiluted samples from each patient were collected in a sterile 5 mL syringe and transferred to a 1.8 mL sterile cryovial (Nunc; Thermo Fisher Scientific, Waltham, MA), and snap-frozen in liquid nitrogen. The cryovial was transferred and stored in a -80 °C freezer until metabolomics analysis.

### Sample preparation and metabolomics analysis

30 µL vitreous sample and 90 µL methanol (4° C) were mixed thoroughly and centrifuged for 10 min at 21,000 RCF and 4° C. A blank sample was made with water instead of vitreous humor. The supernatants were transferred to HPLC vials and analyzed using the global metabolomics method described in Skogvold et al. [18] using the same equipment, solvents, instrumentation and computer software.

Pooled group samples (CTRL, DIA, NPDR and PDR) were made by mixing equal volumes of each sample within each group and analyzed using data dependent MS/MS mode (top 5, stepped normalized collision energy: 20, 50 and 80, dynamic exclusion: 10 s, resolution (m/z 200): 17 500 full width at half maximum, automatic gain control target: 5e5 ion counts and maximum injection time: 100 ms). The pooled quality control sample was made by mixing equal volumes of each group sample and injected between every fifth sample injection to correct for instrumental variation using Full MS mode.

### Data processing and statistics

The LC-MS raw files were processed using Compound Discoverer 3.3 (Thermo Scientific, Waltham, MA, USA) with the workflow template: “Untargeted Metabolomics with Statistics Detect Unknowns with ID using Online Databases and mzLogic”. Principal component analysis (PCA) plots and volcano plots were generated by this workflow. Significance levels for the sample groups were determined by one-way analysis of variance (ANOVA) test followed by posthoc Tukey test using Compund Discoverer 3.3.

### Feature identification

Features with *p*-value < 0.05 and ratio (group x/group y) > 2 or < 0.5 were evaluated and selected for identification based on within-group spread of detected signal, evaluation of chromatograms and feature characteristics matching metabolites of interest.

Identification of the selected features were based on level of confidence in identification, ranked from level 5 to level 1, according to Schymanski et al. [19]. Level 5 is the lowest level of confidence and is reported with only m/z value and retention time of the feature. Level 4 is where a predicted molecular formula (based on MS spectrum) is used to search for match in Chemspider database (http://www.chemspider.com/) and/or Human Metabolome Database (https://hmdb.ca/). Level 3 is suggested candidate(s) based on e.g., class specific fragments in the MS/MS spectrum. Level 2 is where a probable structure based on the MS fragmentation spectrum matches with online databases: mzCloud (https://www.mzcloud.org/), Global Natural Products Social Molecular Networking (https://gnps.ucsd.edu/ProteoSAFe/static/gnps-splash.jsp), MassBank of North America (https://mona.fiehnlab.ucdavis.edu/) and/or Lipid Maps (https://www.lipidmaps.org/). Level 1, the highest level of confidence in identification, is a full match in m/z value, MS fragmentation spectrum and retention time with an in-house library, that at the time of analysis consisted of 491 metabolites analyzed with the same analytical method.

## Results

### Metabolomic signatures

**Fig. 1:**
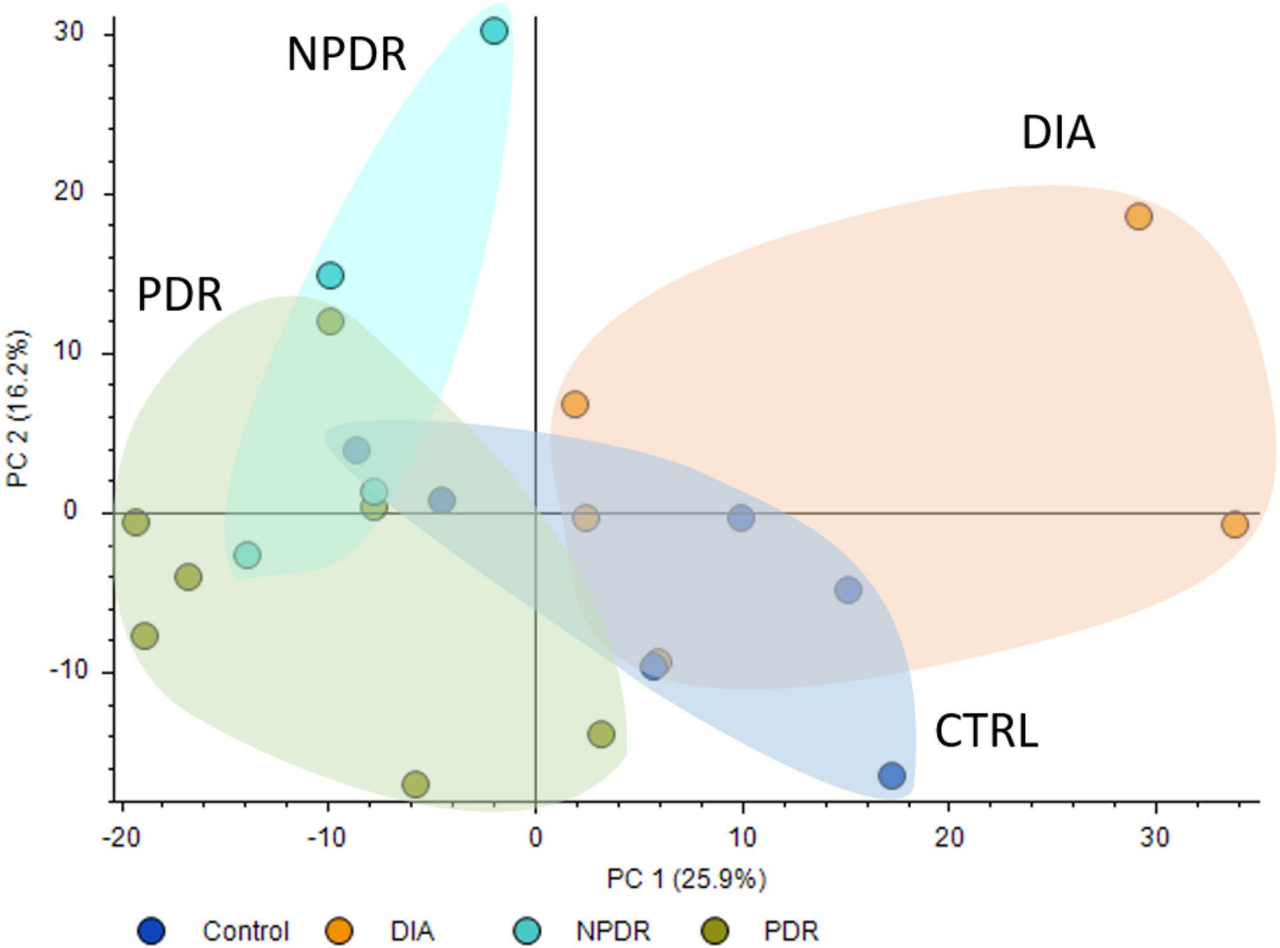
Principle component analysis of the metabolomic profile of the study population. *PC 1 = principal component 1; PC 2 = principal component 2; CTRL = controls without diabetes or retinopathy; DIA = patients with diabetes and no retinopathy; NPDR = patients with non-proliferative diabetic retinopathy; PDR = patients with proliferative diabetic retinopathy*

The study included four groups: CTRL (6 participants), DIA (5 participants), NPDR (5 participants), and PDR (7 participants). One of the patients from the NPDR group was excluded from statistical analysis in the positive ionization mode due to low signal. The PCA plot (Fig. 1) shows clustering patterns among the four groups, indicating differences in the metabolomic profiles between the groups. The DIA group is notably separated from the DR groups. Additionally, the separation between NPDR and PDR groups indicates progressive metabolic alterations as DR advances.

**Fig. 2:**
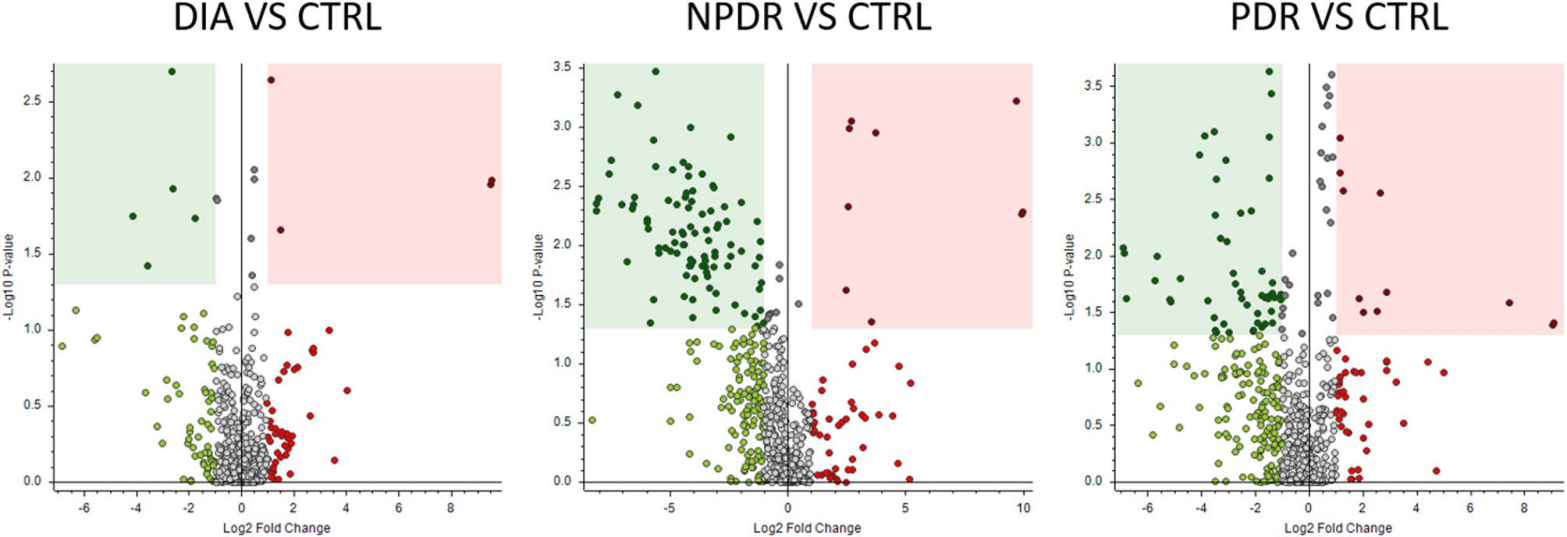
Volcano plots showing differentially abundant metabolites (DAM) between different disease groups (DIA, NPDR and PDR) versus control (CTRL). Colored area indicates DAM’s with statistical significance (*p* < 0.05) and more than doubling in amount (fold change > 2). Dots to the right in each figure represents metabolites that are higher in the respective patient groups than in the controls. Vice versa, dots to the left in each figure represent metabolites that are higher in the controls compared to the respective patients groups. *CTRL = controls without diabetes or retinopathy; DIA = patients with diabetes and no retinopathy; NPDR = patients with non-proliferative diabetic retinopathy; PDR = patients with proliferative diabetic retinopathy*

**Fig. 3:**
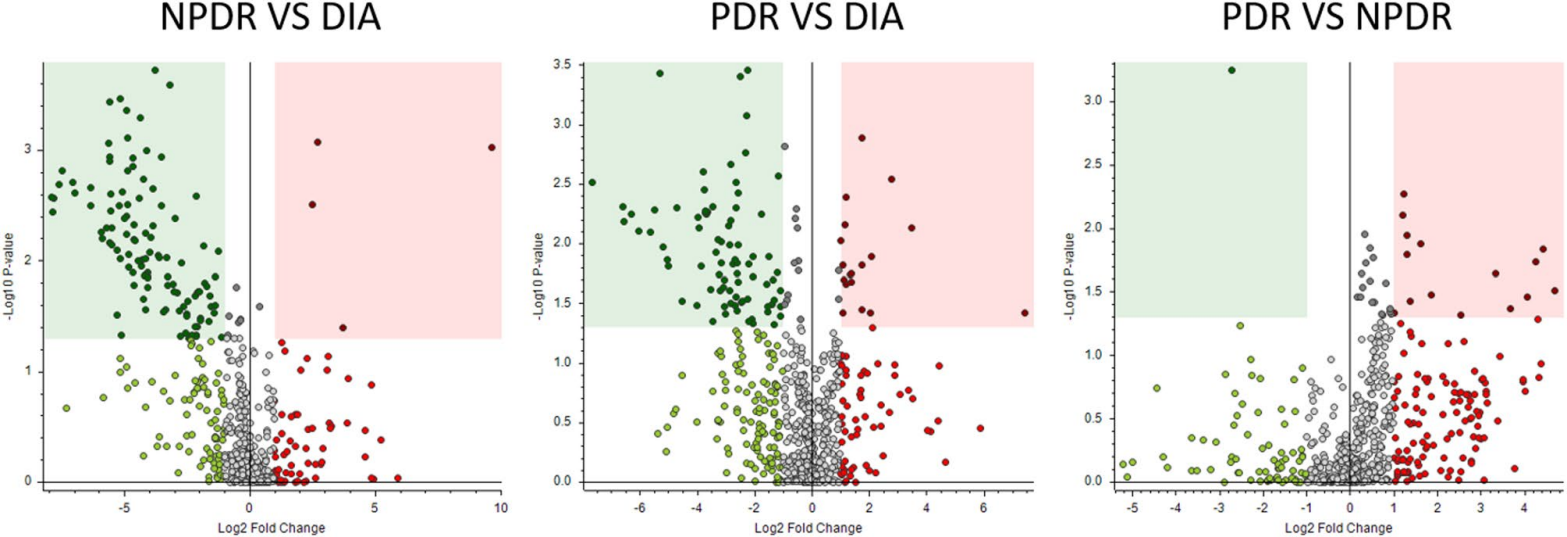
Volcano plots showing differentially abundant metabolites (DAM) between different disease groups (DIA, NPDR and PDR). Colored area indicates DAM’s with statistical significance (*p* < 0.05) and more than doubling in amount (fold change > 2). Dots to the right in each figure represents metabolites that are higher in the groups with more advanced diabetic retinopathy (DR) than in the groups with less advanced disease. Vice versa, dots to the left in each figure represent metabolites that are higher in the groups with less advanced DR compared to the group with more advanced DR. *DIA = patients with diabetes and no retinopathy; NPDR = patients with non-proliferative diabetic retinopathy; PDR = patients with proliferative diabetic retinopathy*

The volcano plots present a visual representation of the number of differentially abundant metabolites (DAM) between disease groups and the controls (Fig. 2) and between the disease groups themselves (Fig. 3). These plots can indicate the degree of metabolic alteration.

In the DIA vs. CTLR plot, significant differences in several metabolites, with some showing higher abundance in the DIA group and others in the CTRL group, was observed. In the NPDR vs. Control and PDR vs. CTRL plots, similar trends as observed in the DIA vs. CTRL comparison was found, indicating progressive metabolic changes moving from diabetes to NPDR and ultimately to PDR.

Between the disease groups, for NPDR vs. DIA, larger significant differences could be observed, suggesting that early retinopathy drastically alters the metabolomic profile from that of diabetes alone. Between the PDR vs. DIA, a further increase in number of significant differences were found, highlighting expanding metabolic changes as retinopathy progresses to advanced stages. Finally, comparing PDR and NPDR, the number of significant differences is lower than comparing DIA with the two DR groups, suggesting a higher degree of metabolic alterations from absence to presence of diabetic retinopathy than from progression of non-proliferative to proliferative retinopathy.

### Differentially abundant metabolites with increasing trends under diabetic retinopathy progression

803 features were detected in positive mode and 533 features were detected in negative mode using global metabolomics. We selected 82 features with *p*-value < 0.05 and ratio (patient/control) > 2 or < 0.5 for further identification (Table S2). After signal grouping, fifteen features were identified at level 1 or 2 of confidence in identification. Ten of these identified molecules showed a significant increase with fold change (FC) > 2 in disease groups.

#### Amino acids and carnitine

**Fig. 4:**
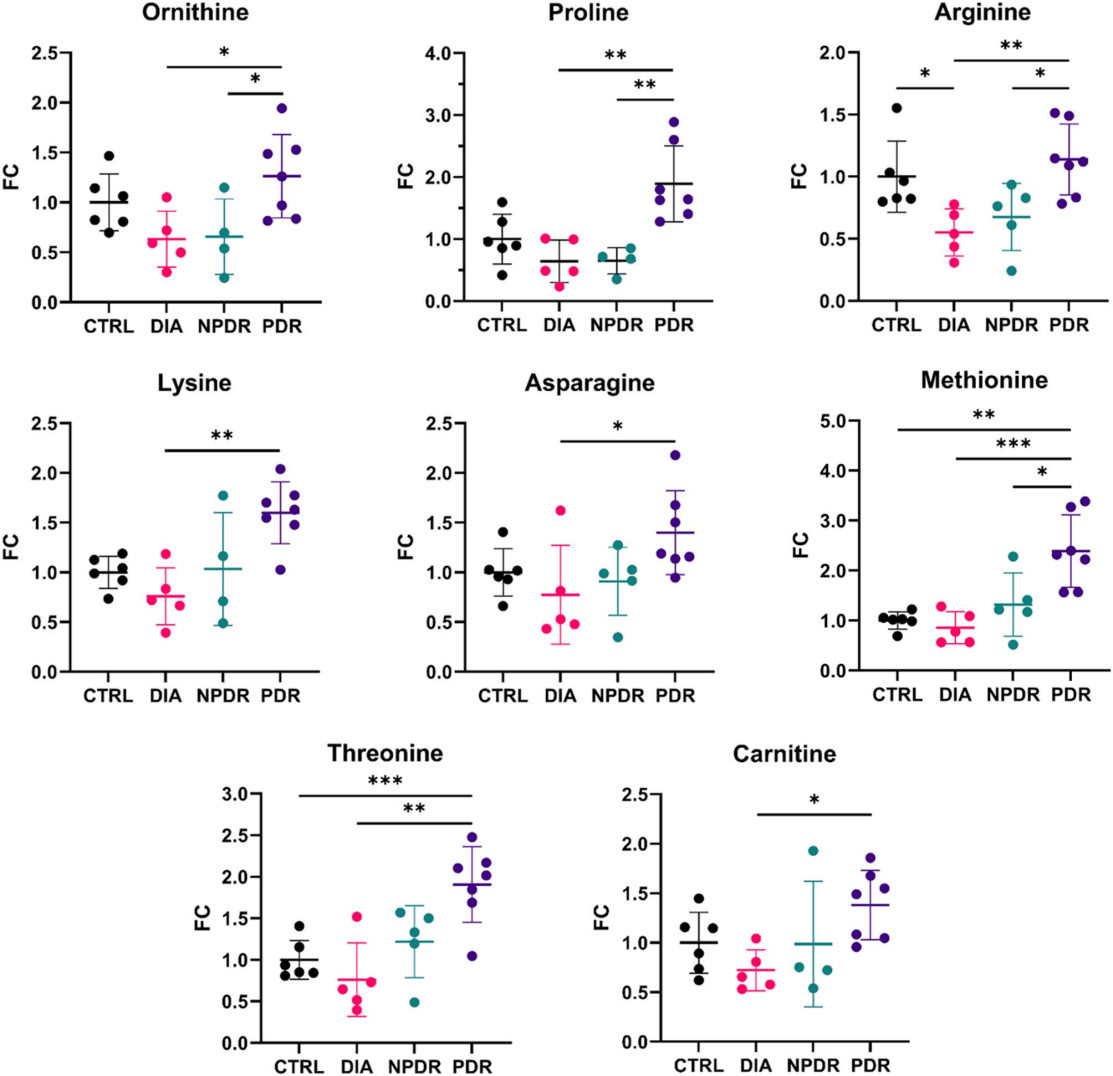
Scatter dot plots showing the trends in levels of molecules in the vitreous of the groups (mean ± SD). FC = fold change vs. control; DIA = patients with diabetes and no retinopathy; NPDR = patients with non-proliferative diabetic retinopathy; PDR = patients with proliferative diabetic retinopathy; *p < 0.05; **p < 0.01; ***p < 0.001

The scatter dot plots show the comparisons of mean FC of molecules between groups, with the mean of the CTRL group as reference (FC = 1) (Fig. 4). Ornithine, proline and arginine were significantly increased in PDR compared to DIA and NPDR, while lysine and asparagine were significantly increased in PDR compared to DIA only. Methionine showed a significant increase in PDR compared to CTRL, DIA and NPDR, and threonine was significantly increased in PDR compared to CTRL and DIA. Additionally, carnitine showed a significant increase in PDR compared to DIA.

#### Drugs

**Fig. 5:**
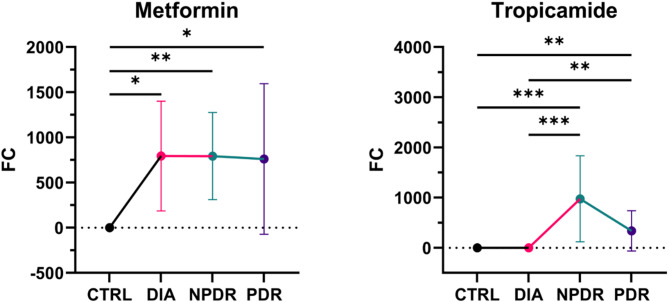
Trendline charts of Metformin and Tropicamide in the vitreous of groups (mean ± SD). FC = fold change vs. control; DIA = patients with diabetes and no retinopathy; NPDR = patients with non-proliferative diabetic retinopathy; PDR = patients with proliferative diabetic retinopathy; *p < 0.05; **p < 0.01; ***p < 0.001

Medications that were administered were detected in the vitreous humor of patients undergoing vitrectomy (Fig. 5). Metformin was detected in all disease groups, but not in controls. Interestingly, Tropicamide was present in the vitreous of patients with NPDR and PDR, but not in CTRL and DIA.

## Discussion

The current pilot study builds on the growing body of research focused on unraveling the metabolomic landscape of DR. Its primary objective was to assess the feasibility of vitreous metabolomic profiling in the context of DR and to generate hypotheses for future investigations. By examining the vitreous metabolome across different stages of the disease, we identified distinct metabolic signatures that may serve as potential biomarkers for both the presence and progression of DR.

The PCA revealed clustering of the CTRL, DIA, NPDR, and PDR groups. This clustering signifies that as DR progresses, the metabolic alterations become more pronounced, particularly between the DIA and DR groups. Our findings are in line with previous studies, which demonstrated significant metabolic differences in DR patients compared to non-diabetic controls [13, 16].

A notable finding in our study was the significant increase in lysine levels in the NPDR and PDR groups compared to controls (*p* < 0.01; Cohen’s *d* = 1.2). Lysine, an essential amino acid involved in proteinogenesis and carnitine production, has been previously reported to be elevated in both serum and vitreous of patients with DR [16, 20, 21]. Lysine’s role in histone modification and its involvement in the formation of advanced glycation end-products such as N-epsilon-carboxymethyl lysine (CML), a known marker of oxidative stress, further underscores its relevance in DR pathogenesis [21–23]. As previously shown, CML has been closely associated with advanced DR stages, supporting our observation of elevated lysine levels in patients with more severe disease [24, 25].

Carnitine, a key player in lipid metabolism, exhibited stage-specific alterations in our study, with significantly higher levels observed in PDR compared to DIA (*p* < 0.05; Cohen’s *d* = 2.1). This aligns with two previous studies that reported elevated carnitine levels in patients with diabetic complications [26, 27]. Carnitine is crucial for transport of long-chain fatty acids, in the form of acylcarnitines, into the mitochondria for β-oxidation, playing a vital role in cellular energy metabolism [28, 29]. Acylcarnitines comprise a wide range of molecules described as biomarkers of mitochondrial function [30]. The observed increase in carnitine in PDR patients suggests that metabolic dysregulation in lipid metabolism may contribute to DR progression [31]. Moreover, previous studies highlighted the association of elevated carnitine levels with neovascularization, a hallmark of PDR, reinforcing its potential as a biomarker for advanced stages of the disease [32].

Proline, a non-essential amino acid involved in collagen synthesis, showed significant increases in NPDR and PDR groups in our study. Previous research has linked elevated proline levels to fibrosis and vascular proliferation in DR [21, 33]. Proline’s role in collagen deposition, coupled with increased arginase activity, suggests that its higher concentration may contribute to fibrovascular scarring, a characteristic feature of PDR [34, 35]. Our findings support the hypothesis that proline metabolism is closely intertwined with DR pathogenesis, particularly in promoting extracellular matrix remodeling and pericyte loss, both of which are critical in DR progression [35–37].

Lysine, carnitine, and proline may serve as biomarkers for the development and progression of DR presents an opportunity for better diagnostics, either as stand-alone biomarkers, or in combination as metabolic ratios for higher specificity and sensitivity. Furthermore, targeted therapies aimed at modulating metabolic pathways that are involved in the pathophysiology, can be developed. For instance, the use of carnitine supplementation has shown promise in improving mitochondrial function and reducing oxidative stress [38]. Additionally, the modulation of arginase activity to regulate proline levels could serve as a therapeutic approach to mitigate fibrosis and vascular damage in advanced DR [33, 39, 40].

Our metabolomic analysis also revealed the presence of drugs, such as metformin and tropicamide, in the vitreous humor of patients undergoing vitrectomy. These findings provide an interesting perspective on how systemic and topical medications penetrate the ocular environment, particularly in the context of DR. The finding of drugs also underlines the power of global metabolomics over targeted analysis. The global approach involves capturing signals of as many molecules as possible in a sample, while a standard targeted approach measures only pre-selected compounds.

Metformin, a first-line drug for managing type 2 diabetes, was detected in all disease groups (DIA, NPDR, and PDR), but, as expected, not in the control group. The presence of metformin in the vitreous humor indicates that this drug, known for its systemic effects on glucose metabolism, also reaches the posterior segment of the eye, indicating increased ocular permeability of systemic drugs in both DM and DR. Previous studies have suggested that metformin may exert protective effects on the retina, possibly through its anti-inflammatory and antioxidant properties [41–43]. Recent evidence also indicates that metformin might reduce oxidative stress and inhibit vascular endothelial growth factor (VEGF) pathways, which are critical in the pathogenesis of PDR [41, 44–46]. Clinical data indicate that metformin users have a lower rate of DR progression​ [47–49]. The observed ability of metformin to reach the retina from systemic circulation, reduce VEGF expression and promote mitochondrial function in patients with diabetes, could explain its beneficial effect on slowing DR progression.

Tropicamide, an anticholinergic agent commonly used to induce pupil dilation during ophthalmic examinations, was also found at higher levels in the NPDR and PDR groups compared to the CTRL and DIA groups. The detection of tropicamide in the vitreous humor is surprising due to its topical use [50]. All patients included in this study received equal amounts of topical tropicamide pre-operatively as a mydriatic for optimal view of the intraocular surgical sight. The elevated drug levels observed in DR patients suggest that topical medications penetrate the vitreous more effectively, potentially entering through the bloodstream via a compromised blood-retinal barrier or directly through the cornea or sclera. Given that blood-retinal barrier dysfunction is a defining feature of DR, its disruption may allow exogenous substances, including medications, to pass from the bloodstream into the vitreous [51–53]. Other mechanisms – such as structural alterations in the vitreous, increased collagen cross-linking, and impaired metabolic clearance – may also contribute to the elevated levels of tropicamide observed in the eyes of patients with DR [54–57]. Alternatively, enhanced transocular penetration could improve the efficacy of future topical treatments for managing DR. In either case, these findings indicate that DR is associated with increased intraocular drug levels.

The strengths of the study are its comprehensive design of analyzing vitreous metabolomes across distinct stages of DR, addressing a critical gap in existing literature. Utilizing advanced HRMS and robust statistical analyses, it identifies stage-specific biomarkers like lysine, carnitine, and proline, offering insights into disease progression and potential for personalized therapeutic strategies. Furthermore, the inclusion of data on ocular drug exposure and clinical applications underscores its novelty and relevance.

While this pilot study provides valuable insights into the metabolomic changes associated with DR progression, there are several limitations to consider. In accordance with previous studies, patients in the CTRL group underwent vitrectomy for macular holes and epiretinal membranes. Consequently, this group does not constitute a true control, as the underlying pathologies may not accurately represent the metabolic profile of a healthy eye. However, performing vitrectomy on individuals without ocular pathology is not ethically or practically feasible. Therefore, the inclusion of patients with macular holes and epiretinal membranes serves as a pragmatic proxy for the healthy ocular metabolome, consistent with established precedents in the literature [13, 15, 16, 58, 59]. Similarly, the patients in the DIA group that were included, underwent surgery for macular holes and symptomatic vitreomacular traction, as it would be unfeasible to operate on an otherwise healthy diabetic eye. Age and duration of diabetes also varied substantially across the study groups. Both factors are known to influence the metabolomic profile, either directly within the vitreous or indirectly through differential exposure to comorbid conditions and therapeutic interventions [60–62]. Consequently, the observed metabolic alterations may, in part, reflect the cumulative systemic metabolic burden associated with prolonged disease duration and aging. To address this limitation, future studies should consider stratifying participants by age and disease duration or employing statistical adjustment methods to control for these confounding variables. Furthermore, the sample size, particularly for the DIA and NPDR groups, was relatively small, which may limit the generalizability and statistical power of our findings. Future studies with larger cohorts are warranted to validate these putative biomarkers and further explore their mechanistic roles in DR pathogenesis. For similar reasons, we did not perform subgroup analyses comparing anti-VEGF–treated patients to anti-VEGF–naïve individuals, as the current sample size lacks sufficient statistical power to support robust conclusions. Future investigations with expanded cohorts should aim to assess the metabolic effects associated with anti-VEGF treatment, as well as the metabolic impact of photocoagulation. Additionally, longitudinal studies tracking metabolic changes over time in DR patients could provide a more comprehensive understanding of how these biomarkers evolve as the disease progresses. Moreover, the vitreous metabolome was analyzed based on the hypothesis that it serves as the most proximate and representative metabolic reservoir reflecting retinal physiological processes. Blood metabolome analysis was not performed in this study and should be included in future metabolomic studies on DR progression to deepen the understanding of the relationship between intravitreal and circulatory metabolic changes. Such studies will enable the comparison of metabolites commonly elevated in the plasma of individuals with diabetes—such as branched-chain amino acids including valine, leucine, and isoleucine—with their corresponding levels in the vitreous humor.

## Conclusions

The present pilot study highlights the potential of metabolomic profiling in identifying stage-specific biomarkers for DR. The alterations in lysine, carnitine, and proline metabolism observed in this study could serve as valuable indicators of disease progression, paving the way for personalized therapeutic strategies aimed at early intervention and improved management of DR. Future studies should aim at validating these findings in more accessible fluids like aqueous humor, tear fluid or serum for early detection of retinopathy development, as well as explore the protective role of metformin within the ocular environment, particularly in preventing the transition from NPDR to PDR. Previous studies on aqueous and vitreous metabolomics have shown that metabolites such as lysine and carnitine are detectable in both tissues [16, 63–65]. We intend to conduct a longitudinal follow-up study with the inclusion of aqueous and serum sampling to investigate dynamic changes in the ocular and blood metabolomes during the progression of DR.

## Electronic supplementary material

Below is the link to the electronic supplementary material.

Supplementary Material 1

Supplementary Material 2

## Data Availability

Data sets used and/or analyzed during the current study are available from the corresponding author on reasonable request.

## Abbreviations

CML: N-epsilon-carboxymethyl lysine
CTRL: Control
DAM: Differentially abundant metabolites
DIA: Diabetes without retinopathy
DM: Diabetes mellitus
DR: Diabetic retinopathy
FC: Fold change
HPLC: High-performance liquid chromatography
HRMS: High-resolution mass spectrometry
NPDR: Optical coherence-tomography
OCT: Optical coherence-tomography
PC: Principal component
PCA: Principal component analysis
PDR: Proliferative diabetic retinopathy
VEGF: Vascular endothelial growth factor

## References

[1] Antonetti DA, Klein R, Gardner TW. Diabetic retinopathy. N Engl J Med. 2012;366(13):1227–39. 22455417 10.1056/NEJMra1005073

[2] Voigt M, Schmidt S, Lehmann T, Köhler B, Kloos C, Voigt U, et al. Prevalence and progression rate of diabetic retinopathy in type 2 diabetes patients in correlation with the duration of diabetes. Exp Clin Endocrinol Diabetes. 2018;126(09):570–6. 29183104 10.1055/s-0043-120570

[3] Lee R, Wong TY, Sabanayagam C. Epidemiology of diabetic retinopathy, diabetic macular edema and related vision loss. Eye Vis. 2015;2(1). 10.1186/s40662-015-0026-2 PMC4657234 26605370

[4] Leasher JL, Bourne RRA, Flaxman SR, Jonas JB, Keeffe J, Naidoo K, et al. Global estimates on the number of people blind or visually impaired by diabetic retinopathy: A Meta-analysis from 1990 to 2010. Diabetes Care. 2016;39(9):1643–9. 27555623 10.2337/dc15-2171

[5] Sabanayagam C, Banu R, Chee ML, Lee R, Wang YX, Tan G, et al. Incidence and progression of diabetic retinopathy: a systematic review. Lancet Diabetes Endocrinol. 2019;7(2):140–9. 30005958 10.1016/S2213-8587(18)30128-1

[6] Safi H, Safi S, Hafezi-Moghadam A, Ahmadieh H. Early detection of diabetic retinopathy. Surv Ophthalmol. 2018;63(5):601–8. 29679616 10.1016/j.survophthal.2018.04.003

[7] Vujosevic S, Aldington SJ, Silva P, Henrnández C, Scanlon P, Peto T, et al. Screening for diabetic retinopathy: new perspectives and challenges. Lancet Diabetes Endocrinol. 2020;8(4):337–47. 32113513 10.1016/S2213-8587(19)30411-5

[8] Laíns Î, Gantner M, Murinello S, Lasky-Su JA, Miller JW, Friedlander M, et al. Metabolomics in the study of retinal health and disease. Prog Retin Eye Res. 2019;69:57–79. 30423446 10.1016/j.preteyeres.2018.11.002

[9] Boulagnon C, Garnotel R, Fornes P, Gillery P. Post-mortem biochemistry of vitreous humor and glucose metabolism: an update. Clin Chem Lab Med CCLM. 2011;49(8):1265–70. 21663468 10.1515/CCLM.2011.638

[10] Midena E, Frizziero L, Midena G, Pilotto E. Intraocular fluid biomarkers (liquid biopsy) in human diabetic retinopathy. Graefes Arch Clin Exp Ophthalmol. 2021. 10.1007/s00417-021-05285-y PMC8589786 34216255

[11] Schrimpe-Rutledge AC, Codreanu SG, Sherrod SD, Mclean JA. Untargeted metabolomics Strategies—Challenges and emerging directions. J Am Soc Mass Spectrom. 2016;27(12):1897–905. 27624161 10.1007/s13361-016-1469-y PMC5110944

[12] Barba I, Garcia-Ramírez M, Hernández C, Alonso MA, Masmiquel L, García-Dorado D, et al. Metabolic fingerprints of proliferative diabetic retinopathy: an 1H-NMR-based metabonomic approach using vitreous humor. Invest Ophthalmol Vis Sci. 2010;51(9):4416–21. 20375324 10.1167/iovs.10-5348

[13] Paris LP, Johnson CH, Aguilar E, Usui Y, Cho K, Hoang LT, et al. Global metabolomics reveals metabolic dysregulation in ischemic retinopathy. Metabolomics Off J Metabolomic Soc. 2016;12:15. 10.1007/s11306-015-0877-5 PMC4651979 26617478

[14] Kunikata H, Ida T, Sato K, Aizawa N, Sawa T, Tawarayama H, et al. Metabolomic profiling of reactive persulfides and polysulfides in the aqueous and vitreous humors. Sci Rep. 2017;7:41984. 28169324 10.1038/srep41984 PMC5294455

[15] Haines NR, Manoharan N, Olson JL, D’Alessandro A, Reisz JA. Metabolomics analysis of human vitreous in diabetic retinopathy and rhegmatogenous retinal detachment. J Proteome Res. 2018;17(7):2421–7. 29877085 10.1021/acs.jproteome.8b00169

[16] Wang H, Fang J, Chen F, Sun Q, Xu X, Lin SH, et al. Metabolomic profile of diabetic retinopathy: a GC-TOFMS-based approach using vitreous and aqueous humor. Acta Diabetol. 2020;57(1):41–51. 31089930 10.1007/s00592-019-01363-0

[17] Zhao T, Wang Y, Guo X, Li H, Jiang W, Xiao Y, et al. Altered Oxylipin levels in human vitreous indicate imbalance in pro-/anti-inflammatory homeostasis in proliferative diabetic retinopathy. Exp Eye Res. 2022;214:108799. 34687725 10.1016/j.exer.2021.108799

[18] Skogvold HB, Sandås EM, Østeby A, Løkken C, Rootwelt H, Rønning PO, et al. Bridging the Polar and hydrophobic metabolome in Single-Run untargeted liquid Chromatography-Mass spectrometry dried blood spot metabolomics for clinical purposes. J Proteome Res. 2021;20(8):4010–21. 34296888 10.1021/acs.jproteome.1c00326 PMC8397434

[19] Schymanski EL, Jeon J, Gulde R, Fenner K, Ruff M, Singer HP, et al. Identifying small molecules via high resolution mass spectrometry: communicating confidence. Environ Sci Technol. 2014;48(4):2097–8. 24476540 10.1021/es5002105

[20] Wang Z, Tang J, Jin E, Zhong Y, Zhang L, Han X, et al. Serum untargeted metabolomics reveal potential biomarkers of progression of diabetic retinopathy in Asians. Front Mol Biosci. 2022;9:871291. 35755823 10.3389/fmolb.2022.871291 PMC9224596

[21] Vidhya S, Ramya R, Coral K, Sulochana KN, Bharathidevi SR. Free amino acids Hydroxyproline, lysine, and glycine promote differentiation of retinal pericytes to adipocytes: A protective role against proliferative diabetic retinopathy. Exp Eye Res. 2018;173:179–87. 29752946 10.1016/j.exer.2018.05.004

[22] Mishra N, Saxena S, Shukla RK, Singh V, Meyer CH, Kruzliak P, et al. Association of serum Nε-Carboxy Methyl lysine with severity of diabetic retinopathy. J Diabetes Complications. 2016;30(3):511–7. 26782022 10.1016/j.jdiacomp.2015.12.009

[23] Ying L, Shen Y, Zhang Y, Wang Y, Liu Y, Yin J, et al. Association of advanced glycation end products with diabetic retinopathy in type 2 diabetes mellitus. Diabetes Res Clin Pract. 2021;177:108880. 34058298 10.1016/j.diabres.2021.108880

[24] Wautier M, Massin P, Guillausseau P, Huijberts M, Levy B, Boulanger E, et al. N(carboxymethyl)lysine as a biomarker for microvascular complications in type 2 diabetic patients. Diabetes Metab. 2003;29(1):44–52. 12629447 10.1016/s1262-3636(07)70006-x

[25] Boehm BO, Schilling S, Rosinger S, Lang GE, Lang GK, Kientsch-Engel R, et al. Elevated serum levels of Nε-carboxymethyl-lysine, an advanced glycation end product, are associated with proliferative diabetic retinopathy and macular oedema. Diabetologia. 2004;47(8):1376–9. 15258735 10.1007/s00125-004-1455-y

[26] Tamamoğullari N, Siliğ Y, İçağasioğlu S, Atalay A. Carnitine deficiency in diabetes mellitus complications. J Diabetes Complications. 1999;13(5):251–3. 10764998 10.1016/s1056-8727(99)00052-5

[27] Liepinsh E, Skapare E, Vavers E, Konrade I, Strele I, Grinberga S, et al. High l-carnitine concentrations do not prevent late diabetic complications in type 1 and 2 diabetic patients. Nutr Res. 2012;32(5):320–7. 22652370 10.1016/j.nutres.2012.03.010

[28] Bremer J. Carnitine–metabolism and functions. Physiol Rev. 1983;63(4):1420–80. 6361812 10.1152/physrev.1983.63.4.1420

[29] Pekala J, Patkowska-Sokola B, Bodkowski R, Jamroz D, Nowakowski P, Lochynski S, et al. L-Carnitine - Metabolic functions and meaning in humans life. Curr Drug Metab. 2011;12(7):667–78. 21561431 10.2174/138920011796504536

[30] McCann MR, De la Rosa G, Rosania MV, Stringer GR. L-Carnitine and acylcarnitines: mitochondrial biomarkers for precision medicine. Metabolites. 2021;11(1):51. 33466750 10.3390/metabo11010051 PMC7829830

[31] Wang WY, Liu X, Gao XQ, Li X, Fang ZZ. Relationship between acylcarnitine and the risk of retinopathy in type 2 diabetes mellitus. Front Endocrinol. 2022;13:834205. 10.3389/fendo.2022.834205 PMC8964487 35370967

[32] Yun JH, Kim JM, Jeon HJ, Oh T, Choi HJ, Kim BJ. Metabolomics profiles associated with diabetic retinopathy in type 2 diabetes patients. PLoS ONE. 2020;15(10):e0241365. 33119699 10.1371/journal.pone.0241365 PMC7595280

[33] Caldwell RB, Zhang W, Romero MJ, Caldwell RW. Vascular dysfunction in retinopathy-an emerging role for arginase. Brain Res Bull. 2010;81(2–3):303–9. 19737603 10.1016/j.brainresbull.2009.08.025 PMC2815222

[34] Roy S, Maiello M, Lorenzi M. Increased expression of basement membrane collagen in human diabetic retinopathy. J Clin Invest. 1994;93(1):438–42. 8282817 10.1172/JCI116979 PMC293808

[35] Roy S, Amin S, Roy S. Retinal fibrosis in diabetic retinopathy. Exp Eye Res. 2016;142:71–5. 26675403 10.1016/j.exer.2015.04.004 PMC4683353

[36] Du J, Zhu S, Lim RR, Chao JR. Proline metabolism and transport in retinal health and disease. Amino Acids. 2021;53(12):1789–806. 33871679 10.1007/s00726-021-02981-1 PMC8054134

[37] Resnikoff HA, Miller CG, Schwarzbauer JE. Implications of fibrotic extracellular matrix in diabetic retinopathy. Exp Biol Med. 2022;247(13):1093–102. 10.1177/15353702221087175 PMC9335512 35410521

[38] Cao Y, Li X, Shi P, Wang L, xin. Sui Z Guo. Effects of L-carnitine on high glucose-induced oxidative stress in retinal ganglion cells. Pharmacology. 2014;94(3–4):123–30. 25247444 10.1159/000363062

[39] Shosha E, Fouda AY, Narayanan SP, Caldwell RW, Caldwell RB. Is the arginase pathway a novel therapeutic avenue for diabetic retinopathy?? J Clin Med. 2020;9(2):425. 32033258 10.3390/jcm9020425 PMC7073619

[40] Narayanan SP, Rojas M, Suwanpradid J, Toque HA, Caldwell RW, Caldwell RB. Arginase in retinopathy. Prog Retin Eye Res. 2013;36:260–80. 23830845 10.1016/j.preteyeres.2013.06.002 PMC3759622

[41] Li Y, Gappy S, Liu X, Sassalos T, Zhou T, Hsu A, et al. Metformin suppresses pro-inflammatory cytokines in vitreous of diabetes patients and human retinal vascular endothelium. PLoS ONE. 2022;17(7):e0268451. 35802672 10.1371/journal.pone.0268451 PMC9269956

[42] Zou AL, He T, Chen J, Sun X, Fan D. X, Rescue of Retinal Degeneration in rd1 Mice by Intravitreally Injected Metformin. Front Mol Neurosci. 2019 Apr 26 [cited 2024 Oct 13];12. Available from: https://www.frontiersin.org/journals/molecular-neuroscience/articles/10.3389/fnmol.2019.00102/full 10.3389/fnmol.2019.00102 PMC6497809 31080404

[43] Amin SV, Khanna S, Parvar SP, Shaw LT, Dao D, Hariprasad SM, et al. Metformin and retinal diseases in preclinical and clinical studies: insights and review of literature. Exp Biol Med. 2022;247(4):317–29. 10.1177/15353702211069986 PMC8899338 35068220

[44] Hsu SK, Cheng KC, Mgbeahuruike MO, Lin YH, Wu CY, Wang HMD, et al. New insight into the effects of Metformin on diabetic retinopathy, aging and cancer: nonapoptotic cell death, immunosuppression, and effects beyond the AMPK pathway. Int J Mol Sci. 2021;22(17):9453. 34502359 10.3390/ijms22179453 PMC8430477

[45] Zhang K, Wang T, Sun GF, Xiao JX, Jiang LP, Tou FF, et al. Metformin protects against retinal ischemia/reperfusion injury through AMPK-mediated mitochondrial fusion. Free Radic Biol Med. 2023;205:47–61. 37253410 10.1016/j.freeradbiomed.2023.05.019

[46] Yi QY, Deng G, Chen N, Bai ZS, Yuan JS, Wu GH, et al. Metformin inhibits development of diabetic retinopathy through inducing alternative splicing of VEGF-A. Am J Transl Res. 2016;8(9):3947–54. 27725874 PMC5040692

[47] Li Y, Ryu C, Munie M, Noorulla S, Rana S, Edwards P, et al. Association of Metformin treatment with reduced severity of diabetic retinopathy in type 2 diabetic patients. J Diabetes Res. 2018;2018(1):2801450. 29854819 10.1155/2018/2801450 PMC5952500

[48] Fan YP, Wu CT, Lin JL, Hsiung CA, Liu HY, Lai JN, et al. Metformin treatment is associated with a decreased risk of nonproliferative diabetic retinopathy in patients with type 2 diabetes mellitus: A Population-Based cohort study. J Diabetes Res. 2020;2020(1):9161039. 32377525 10.1155/2020/9161039 PMC7189314

[49] Gerardo González-González J, Cesar Solis R, Díaz González-Colmenero A, Raygoza-Cortez K, Moreno-Peña PJ, Sánchez AL, et al. Effect of Metformin on microvascular outcomes in patients with type 2 diabetes: A systematic review and meta-analysis. Diabetes Res Clin Pract. 2022;186:109821. 35247521 10.1016/j.diabres.2022.109821 PMC9064963

[50] Yepez J, De JC, Arevalo JF. Topical Anesthesia In Posterior Vitrectomy. Retina. 2000;20(1):41. 10696746 10.1097/00006982-200001000-00008

[51] Rudraraju M, Narayanan SP, Somanath PR. Regulation of blood-retinal barrier cell-junctions in diabetic retinopathy. Pharmacol Res. 2020;161:105115. 32750417 10.1016/j.phrs.2020.105115 PMC7755666

[52] Pfeiffer A, Spranger J, Meyer-Schwickerath R, Schatz H. Growth factor alterations in advanced diabetic retinopathy: A possible role of blood retina barrier breakdown. Diabetes. 1997;46(Supplement_2):S26–30. 9285495 10.2337/diab.46.2.s26

[53] Frey T, Antonetti DA. Alterations to the Blood–Retinal barrier in diabetes: cytokines and reactive oxygen species. Antioxid Redox Signal. 2011;15(5):1271–84. 21294655 10.1089/ars.2011.3906

[54] Sebag J, Buckingham B, Charles MA, Reiser K. Biochemical abnormalities in vitreous of humans with proliferative diabetic retinopathy. Arch Ophthalmol. 1992;110(10):1472–6. 1417549 10.1001/archopht.1992.01080220134035

[55] Gao BB, Chen X, Timothy N, Aiello LP, Feener EP. Characterization of the vitreous proteome in diabetes without diabetic retinopathy and diabetes with proliferative diabetic retinopathy. J Proteome Res. 2008;7(6):2516–25. 18433156 10.1021/pr800112g

[56] Semeraro F, Cancarini A, dell’Omo R, Rezzola S, Romano MR, Costagliola C. Diabetic retinopathy: vascular and inflammatory disease. J Diabetes Res. 2015;2015(1):582060. 26137497 10.1155/2015/582060 PMC4475523

[57] Chernykh VV, Varvarinsky EV, Smirnov EV, Chernykh DV, Trunov AN. Proliferative and inflammatory factors in the vitreous of patients with proliferative diabetic retinopathy. Indian J Ophthalmol. 2015;63(1):33. 25686060 10.4103/0301-4738.151464 PMC4363955

[58] Tomita Y, Cagnone G, Fu Z, Cakir B, Kotoda Y, Asakage M, et al. Vitreous metabolomics profiling of proliferative diabetic retinopathy. Diabetologia. 2021;64(1):70–82. 33099660 10.1007/s00125-020-05309-y PMC7718434

[59] Wang H, Li S, Wang C, Wang Y, Fang J, Liu K. Plasma and vitreous metabolomics profiling of proliferative diabetic retinopathy. Invest Ophthalmol Vis Sci. 2022;63(2):17. 35133401 10.1167/iovs.63.2.17 PMC8842420

[60] Lau CHE, Manou M, Markozannes G, Ala-Korpela M, Ben-Shlomo Y, Chaturvedi N, et al. NMR metabolomic modeling of age and lifespan: A multicohort analysis. Aging Cell. 2024;23(7):e14164. 38637937 10.1111/acel.14164 PMC11258446

[61] Castro A, Signini ÉF, De Oliveira JM, Di Medeiros Leal MCB, Rehder-Santos P, Millan-Mattos JC, et al. The aging process: A metabolomics perspective. Molecules. 2022;27(24):8656. 36557788 10.3390/molecules27248656 PMC9785117

[62] ‘t Hart LM, Vogelzangs N, Mook-Kanamori DO, Brahimaj A, Nano J, van der Heijden AAWA, et al. Blood metabolomic measures associate with present and future glycemic control in type 2 diabetes. J Clin Endocrinol Metab. 2018;103(12):4569–79. 30113659 10.1210/jc.2018-01165

[63] Zelentsova EA, Yanshole LV, Melnikov AD, Kudryavtsev IS, Novoselov VP, Tsentalovich YP. Post-mortem changes in metabolomic profiles of human serum, aqueous humor and vitreous humor. Metabolomics. 2020;16(7):80. 32613532 10.1007/s11306-020-01700-3

[64] Lillo A, Marin S, Serrano-Marín J, Binetti N, Navarro G, Cascante M et al. Targeted Metabolomics Shows That the Level of Glutamine, Kynurenine, Acyl-Carnitines and Lysophosphatidylcholines Is Significantly Increased in the Aqueous Humor of Glaucoma Patients. Front Med. 2022 Jul 22 [cited 2025 Apr 23];9. Available from: https://www.frontiersin.orghttps://www.frontiersin.org/journals/medicine/articles/10.3389/fmed.2022.935084/full 10.3389/fmed.2022.935084 PMC9354463 35935793

[65] Jin H, Zhu B, Liu X, Jin J, Zou H. Metabolic characterization of diabetic retinopathy: an 1H-NMR-based metabolomic approach using human aqueous humor. J Pharm Biomed Anal. 2019;174:414–21. 31212142 10.1016/j.jpba.2019.06.013

