# Supplementary material for: Metabolomic biomarkers in vitreous humor: unveiling the molecular landscape of diabetic retinopathy progression
Source: Int J Retina Vitreous. 2025 May 22;11:58. doi: 10.1186/s40942-025-00682-5 (PMC12096489; doi:10.1186/s40942-025-00682-5)
Supplement: Supplementary file 1 — Supplementary Material 1 [file 40942_2025_682_MOESM1_ESM.pdf]

Table S1. Indications for Vitrectomy.

CTRL = controls without diabetes or retinopathy; DIA = patients with diabetes and no retinopathy; NPDR = patients with non-proliferative diabetic retinopathy; PDR = patients with proliferative diabetic retinopathy; DM = diabetes mellitus; VEGF = vascular endothelial growth factor.

INDICATION	CTRL	DIA	NPDR	PDR
**Macular hole**	3	2	0	0
**Epiretinal membrane**	3	0	2	4
**Symptomatic vitreomacular traction**	0	3	1	2
**Non-clearing vitreous opacities**	0	0	1	1
